# Expressed sequence tags (ESTs) and simple sequence repeat (SSR) markers from octoploid strawberry *(Fragaria *× *ananassa)*

**DOI:** 10.1186/1471-2229-5-12

**Published:** 2005-06-28

**Authors:** Kevin M Folta, Margaret Staton, Philip J Stewart, Sook Jung, Dawn H Bies, Christopher Jesdurai, Dorrie Main

**Affiliations:** 1 Plant Molecular and Cellular Biology Program and Horticultural Sciences Department, University of Florida, Gainesville, FL, USA; 2 Genetics, Biochemistry & Life Science Studies, Clemson University, Clemson, SC, USA

## Abstract

**Background:**

Cultivated strawberry (*Fragaria *× *ananassa*) represents one of the most valued fruit crops in the United States. Despite its economic importance, the octoploid genome presents a formidable barrier to efficient study of genome structure and molecular mechanisms that underlie agriculturally-relevant traits. Many potentially fruitful research avenues, especially large-scale gene expression surveys and development of molecular genetic markers have been limited by a lack of sequence information in public databases. As a first step to remedy this discrepancy a cDNA library has been developed from salicylate-treated, whole-plant tissues and over 1800 expressed sequence tags (EST's) have been sequenced and analyzed.

**Results:**

A putative unigene set of 1304 sequences – 133 contigs and 1171 singlets – has been developed, and the transcripts have been functionally annotated. Homology searches indicate that 89.5% of sequences share significant similarity to known/putative proteins or Rosaceae ESTs. The ESTs have been functionally characterized and genes relevant to specific physiological processes of economic importance have been identified. A set of tools useful for SSR development and mapping is presented.

**Conclusion:**

Sequences derived from this effort may be used to speed gene discovery efforts in *Fragaria *and the Rosaceae in general and also open avenues of comparative mapping. This report represents a first step in expanding molecular-genetic analyses in strawberry and demonstrates how computational tools can be used to optimally mine a large body of useful information from a relatively small data set.

## Background

Commercial strawberry has a value of 1.4 billion dollars in the United States, and represents a significant regional crop throughout the world. Despite its value, fewer than 100 annotated sequences existed in public databases in early 2004. The information discrepancy is a consequence of limited molecular study in the challenging octoploid cultivated varieties. The thin public informatics base hence represents a barrier to meaningful study of functional genomics, genetic mechanisms, as well as the molecular-systematic relationships between the octoploid strawberry, the Rosaceae and other species. The lack of basic sequence information hinders the development of transgenic technologies that would advance molecular-physiological studies and potentially benefit the grower and consumer. Overall, the dearth of sequence information has limited agile molecular resolution studies in this important crop plant.

To remedy this discrepancy ~1800 expressed sequence tags (ESTs) were sequenced from a whole-plant cDNA library derived from various tissues of the Strawberry Festival cultivar. This cultivar was chosen because of its east-coast and west-coast lineage as well as its range of favorable horticultural attributes. 'Strawberry Festival' produces large, uniform, firm fruit, and is resistant to *Botrytis cinera*, the causative agent behind gray mold [1]. It is a predominant cultivar grown in Florida, and has been well studied in many reports of fungicide use, disease resistance and post-harvest fruit quality. The study of an important commercial variety will provide tools to directly aid breeding and probe genetic mechanisms in these cultivars.

Strawberry has unrealized potential as a research model and tool, and the lack of molecular markers for breeding and the eventual need for genetic improvement of the current suite of cultivars makes sequence examination especially timely. Information gained from the octoploid will also translate to defining molecular markers to facilitate mapping in both the diploid species (*eg*. *Fragaria vesca *and *Fragaria nubicola*) as well as octoploid cultivars. A strong sequence database is the cornerstone of functional genomics studies, and this information will aid development of such tools in *Fragaria *and in the Rosaceae in general. Definition of expressed gene sequence variation in the octoploid may aid in the understanding of polyploid evolution and/or silencing of component genomes. Sequence information constitutes a basis for eventual reverse-genetic and activation-tag studies. Both the diploid and octoploid species are excellent candidates for such studies as they are efficiently transformed and regenerated [2-4], possess a diploid genome that is slightly larger than that of *Arabidopsis thaliana *[5], and can be rapidly propagated from seed (3–5 months) or runners [6]. Strawberry also may be an excellent candidate as a bioreactor, a system to manufacture specific compounds of interest. A presentation of the elements of the strawberry transcriptome facilitates the initiation of such studies.

Despite strawberry's crop value and potential as a research tool, a formal analysis of EST data has not been reported. In this report we identify over 1300 unique transcripts assembled from 1,847 ESTs derived from whole-plant vegetative tissues 24 h after salicylic acid treatment. The cDNA library was prepared from total RNA pooled from roots, petioles, stolons, leaves and meristems to generate a diverse set of transcripts with limited redundancy. Multiple analyses, such as developing a unigene set, annotation with putative function and identification of SSRs, opens additional paths that will speed research into strawberry physiology, evolution, genetics and genomics. This represents the first major EST report from *Fragaria *and can now serve as a baseline for these further studies.

## Results

### The *Fragaria *× *ananassa *EST library

The Lambda ZAP cDNA library was generated from whole-plant tissues from mature plants 24 after salicylic acid treatment. The details of salicylate treatment, plant materials and library construction are presented in Methods.

### EST processing and assembly

A total of 1847 of ESTs were sequenced, resulting in 1505 high-quality trimmed sequences which were submitted to GenBank on August 6, 2004. Representing a success rate of 81.5%, these sequences have an average length of 613 bp and a PHRED quality value of 35. Assembly of the sequences into a unigene was performed in order to reduce redundancy of the sequences and identify those coding for the same protein (Methods, Assembly). The total unigene consists of 1171 singlets for a total of 1304 unigenes.

Contigs were assembled from EST sequences. The final unigene has 133 contigs, 120 comprised of two or three merged ESTs. Eight contigs were assembled from four individual ESTs. Contigs assembled from five or more ESTs may be useful to deconstruct in the interest of studying allelic diversity in the octoploid. In diploid species alleles represent heterozygousity at a given locus as well as gene duplication and subfunctionalization of a given coding region. Allelic diversity is potentially enriched in the octoploid, since the octoploid maintains the alleles maintained from at least three donor diploid genomes. Expression of specific alleles may be informative, as patterns may be traced back to the diploid genome contributors, allowing description of expression from within, or between, donor genomes.

For instance, Contig 23 represents *psaL*, a nuclear-encoded subunit of the photosystem 1 reaction center. The contig was assembled from five ESTs, two of which (4C07 and 6C09) are identical in sequence yet vary in length. The other members contain SNPs, especially 18C04, which maintains five unique base changes over a 540 bp alignment of all five ESTs. Others contain a single alteration in this relatively conserved gene sequence. Similar results were observed for contigs 32 and 99, which were assembled from seven and nine ESTs, respectively.

Other contigs have been assembled from many ESTs, such as Contigs 29 and 12. These contigs encode light-harvesting, chlorophyll-binding (*Lhcb*, formerly *cab*) proteins and a non-specific lipid transfer protein, respectively. The ESTs corresponding to these genes arise from small multigene families within a diploid genome in most species, making these constructs less useful for studying between-genome polymorphisms.

### Functional annotation

Computational tools are now regularly used to infer function based upon significant sequence similarity toexperimentally verifiedproteins or putative proteins. These analyses implement FASTA and BLAST comparisons against non-redundant databases as well as GO annotation. The EST sequences were compared against known databases using these tools.

Protein homology searches were performed in order to identify the putative function of the ESTs (Methods, Functional Characterization). NCBI's non-redundant (nr) protein and Rosaceae EST databases searches were run on February 27, 2005 using the FASTX3.4 algorithm [7]. The nr database contained 2321663 protein amino acid sequences at the time of the search. Of the 1304 unigenes, 1105, or 84.74% of the set, had significant matches to this database (Table 1). A comparison against SWISS-PROT was performed on July 26, 2004, yielding a lower number of significant matches. SWISS-PROT is a curated, highly-annotated, smaller database of 153,871 proteins of demonstrated function. 714 of the unigenes (54.75%) had significant matches (Table 1). Only 191 of the unigenes did not match a protein in either of these two protein databases (Table 2). Upon close scrutiny the EST sequences did not contain significant open reading frames, suggesting that the EST sequence represents long untranslated regions, structural RNAs, or *bona fide *proteins, unique to *Fragaria *based on current comparisons.

### Comparisons to Rosaceae ESTs

Table 1 also presents the results of comparison of the unigene against publicly-available Rosaceae ESTs in order to assess how *Fragaria *relates to the rest of the Rosaceae family at the gene sequence and content levels. The BLASTN algorithm was then used for EST homology searches against known Rosaceae ESTs. 227,250 Rosaceae ESTs were downloaded from dbEST. Of the 1304 unigenes, 835 (64.03%) had significant homology to other Rosaceae ESTs. Since this dataset is composed of public ESTs, it contains a large amount of redundancy. The majority of public ESTs have been sequenced from the 5' end, so ESTs generated from the 3' end may be less likely to find homologs in a search against public ESTs. Still, of the 191 ESTs that did not show significant homology with SWISS-PROT and Genbank nr (Table 2), 54 ESTs had homologs represented in the Rosaceae EST set. This leaves 137 transcripts that show no significant homology outside of *Fragaria *within the Rosaceae family. These ESTs were compared against the TIGR plant repeat databases to test if they may have originated from retroelement expression. None of the apparently *Fragaria*-specific transcripts exhibited significant homology with sequences within the repeat database.

### Characterization by gene ontology

**Figure 1:**
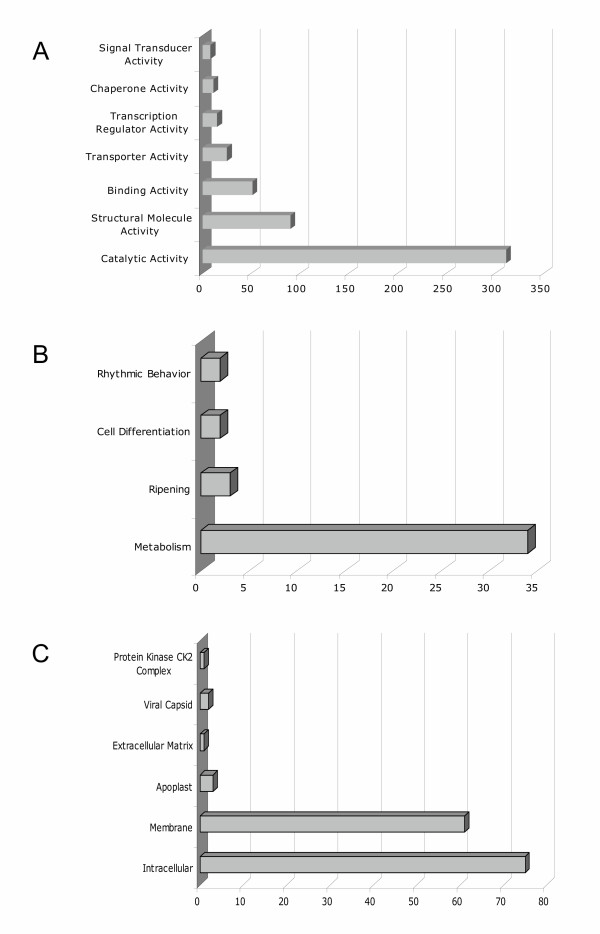
**Strawberry EST characterization as derived from GO analyses**. ESTs were assigned to GO categories based onA) Functional GO matches B) Process GO matches C) Component GO matches.

The *Fragaria *unigenes were further annotated by gene ontology (GO) assignment based on the single "best hit" match against the SWISS-PROT database. All 714 ESTs with hits to SWISS-PROT have matching GO-Terms (Figure 1). The three categories are function (Panel A), process (Panel B), and component (Panel C). For molecular function, the strawberry ESTs were assigned to eight categories. The majority (51%) of the ESTs were assigned to "Catalytic Activity" (GO:0003824). For biological process, the ESTs were assigned to four categories with the majority (77%) representing genes participating in metabolism (GO: 0008152). When grouped according to likely cellular component, the ESTs were assigned to six categories and 93% were covered by two GO terms: "Intracellular" (GO:0005622) and "Membrane" (GO:0016020). The full chart of the assignment of EST's to specific GO Term categories may be viewed on the GDR website [8].

### Homology to mapped peach ESTs

Linkage relationships have been identified for many peach ESTs and have facilitated placement on the peach genetic map. Comparison of the Fragaria unigene to this set of ESTs presents a basis for developing linkage relationships between the established peach, and growing *Fragaria*, linkage maps. A series of peach ESTs have been conclusively anchored to genetic maps by sharing BACs with genetic markers previously used for BAC hybridization [9]. Of the 295 mapped peach ESTs 22 (7.04%) showed a significant match with the strawberry unigene (Table 3).

### Computational analysis for SSRs and ORFs in the ESTs

Simple Sequence Repeats (SSRs) were identified in the strawberry unigene data set (Open Reading Frame and Microsatellite Analysis, Methods). In this study, SSRs are defined as dimers with at least 5 repeats, trimers with at least 4 repeats, tetramers with at least 3 repeats, and pentamers with at least 3 repeats. 190 unigene sequences (14%) were found to have one or more repeat, and 79 different motifs were identified within the set of clones. A total of 269 SSRs were found with trimers being the most common motif length (Table 4). The frequency of motifs for all the possible dimers and trimers is listed in Table 5. To examine the distribution of SSRs in the putative coding region and the UTR, we detected open reading frames in the unigenes using the FLIP program (Brossard 1997). When the longest open reading frame was selected as the putative coding region, 176 (65.4%) of these microsatellites were found inside putative coding regions. When filtered for the most optimal primer candidates (40–60% GC content) a total of 208 SSR-flanking sequences met the criteria (Table 6). These optimal candidates can be downloaded via the GDR ftp site [10].

## Discussion

*Fragaria *× *ananassa *is complex polyploid, arising from a spontaneous cross between *Fragaria virginiana *and *Fragaria chiloensis*. The genome contains contributions from at least three diploid species [11,12]. Over the past century cultivation of octoploid strawberry has progressed solely on the careful efforts of breeders, physiologists and biochemists. This complex genome and coincident awkward genetics has slowed the development of molecular markers and other tools that would benefit breeding efforts and understanding of strawberry genomics. This report details a starting point to advance the traditional strawberry research avenues using modern molecular tools to forward structural-and functional-genomics studies in this important crop species. As important, it demonstrates that computational tools may be used to comprehensively mine large quantities of important data from a relatively small data set. As these tools become available as web-based applications, small sequencing efforts may extract valuable information that may shape research questions in under-represented crops like strawberry.

Recent efforts demonstrate the importance of sequence information as the basis of functional-genomics studies. Previous reports of gene expression in strawberry have been dependent on the discovery and characterization of specific genes of interest, such as an O-methyltransferase associated with flavor [13], enzymes that influence fruit firmness [14-17], as well as several others [18,19]. Current technologies have the capacity to assess genome-wide transcriptome changes associated with a given treatment or developmental process [20,21]. Recent studies in cultivated strawberry have implemented proprietary sequence information in a microarray format to unveil the transcriptome that coincides with fruit ripening [22-24]. These studies have identified critical regulators of fruit flavor. The first study identified strawberry alcohol acetyl transferase as a critical enzyme in the production of volatiles esters. The associated transcript increased during fruit ripening and the recombinant protein catalyzes the appropriate syntheses from a variety of substrates in *E. coli *[23]. A recent functional-genomics study characterized *Nerolidol Synthase 1*, the enzyme that catalyzes the formation of the flavor compounds linalool and nerolidol from geranyl diphosphase and farnesyl diphosphate, respectively. The enzyme is expressed in the receptacle of ripening fruit, not in leaves, and is highly expressed in cultivated species relative to wild species. The report concludes that selection of cultivated varieties for fruit flavor fixed mechanisms to express and localize terpene-associated enzymes that favorably affected flavor, while repressing those that make fruits less desirable [22]. Although the factors leading to fruit flavor in strawberry have been studied for decades, a transcriptome survey produced the most definitive results, owing again to the usefulness of a sequence database in *Fragaria*.

The transcripts characterized from this project will allow development of genomics resources for the study of other important physiological responses. A subset of these ESTs is shown in Table 7. These ESTs are relevant to processes of interest to the strawberry industry and may represent important molecular tools to researchers. The first set represents a series of ESTs with sequence homology to genes associated with the photoperiodic control of flowering. These include close homologs to CONSTANS (CO), a likely transcription factor that induces specific meristem identity genes under the appropriate photoperiod [25,26]. A homolog of a critical regulator of meristem identity AGL20/SUPPRESSOR OF CO OVEREXPRESSION was also identified. This gene encodes a MADS-box transcription factor that likely functions downstream of CO in conferring light signals to the promoters of meristem identity genes [27]. An EST representing VERNALIZATION INSENSITIVE 3 also was identified in this library. VIN3 is a protein shown to function downstream of CO in regulating seasonal flowering responses [28]. VIN3 is a chromatin-remodeling protein that represses FLC, a protein that negatively-regulates CO function [29] allowing the plant to appropriately time flowering relative to seasonal chilling.

Analysis of this dataset revealed a suite of likely homologs to pathogenesis-related (PR) genes, such as thionins, *Ndr1*, β 1-3-glucanase and chitinases, and LRR proteins. The prevalence of this family of proteins was not surprising as the plants were treated with salicylic acid 24 h before RNA harvest to enrich for PR genes in the library. These genes are of particular interest to plant scientists because of their potential to help define the mechanism(s) of disease resistance and susceptibility. It is possible that these genes may be especially useful targets for antisense or overexpression in unveiling these agriculturally-important traits, or possibly in the design of transgenic plants with heightened resistance to common plant pathogens. All of these facets are important, as strawberry cultivation requires copious application of fungicides and/or bacteriostatic compounds to ensure proper fruit set.

Of interest to this laboratory are homologs of genes associated with photomorphogenesis, such as *Hy5 *and *Non-phototropic hypocotyl 3*. These both play roles in early light development, yet HY5 also has been shown to influence downstream developmental processes such as fruit ripening and pigmentation [30] and also binds to the promoters of genes associated with circadian clock progression [31].

The information distilled from all of these analyses can now be used to design strawberry-specific probes to assess gene expression patterns and develop transgenics to directly test gene function. These important studies are underway and will facilitate comparisons between the biological sensory/response mechanisms in strawberry to those of model systems.

The apparent sequence conservation between *Fragaria *and other rosaceous tree crops suggests that cross-species microarray studies may be productive within the Rosaceae. This study demonstrates that less than 11% of the ESTs are unique to strawberry. This value is likely inflated, as ESTs by nature contain variable untranslated regions and other features that may preclude efficient identification of homologs. Of the 1305 ESTs, 835 have strong homology with other Rosaceae ESTs. Those featuring over 85% homology over 100 bases are between 86 and 100% identical to transcripts isolated from other Rosaceae, with an average identity of 91% (+/-0.001%). The high degree of similarity may be a useful platform for comparisons between molecular-mechanistic differences exhibited between diverse species with little sequence variation. Here, the diversity within the Rosaceae is likely due to variation in gene expression, and EST data and microarray technologies are an idea platform to study these patterns.

The relatively extensive genetic mapping in *Prunus *has delineated linkage associations between genes of the genus, those in select Rosaceae species and even Arabidopsis [32]. Physical maps have also been developed from transcript mapping [9]. The ESTs from this *Fragaria *collection were compared to the mapped peach genes, and 23 agreed with strong homology (Table 3). These relationships are important as they present the basis to study structural relationships between cousin species within the Rosaceae. Since these loci are mapped in peach, they represent excellent loci to also add to the growing diploid strawberry linkage map [33], and eventually map in the octoploid.

Mapping efforts may also be hastened from identification of SSRs. SSRs derived from ESTs provide a basis to assign linkage relationships to known gene products, and such studies have been initiated in diploid strawberry [33]. In the EST collection presented herein, a number of SSRs are present in transcripts correlating to putative allergens, regulators of the circadian clock, and general housekeeping genes. These transcripts can now be readily mapped in the diploid using existing populations, and such studies are currently underway. Furthermore, specific genes of interest can be studied for variation within diploid species or for intron-specific polymorphisms that will allow their assignment to the diploid strawberry linkage map. These studies will ultimately facilitate the generation of molecular markers to follow traits/genes of interest in the commercial cultivars, adding the resolution of molecular tools to complement conventional breeding strategies.

The general proportions of the different functional groups (Figure 1) reflect well the expected state of the mature plant transcriptome as reported in previous studies. Transcripts encoding enzymes associated with the cell cycle, cytoskeleton or cell walls are not abundant as mature plants are less reliant on processes governing greater cell number or cell size. Approximately half of the transcripts associated with photosynthesis are members of the chlorophyll a/b binding protein family; the other half typically contains plastid-encoded transcripts. As expected, the majority of transcripts detected represent enzymes of general metabolism.

## Conclusion

Although a small EST set, the complete suite of analyses performed herein demonstrate that a finite transcriptome snapshot may provide ample resources to seed additional study. Here a relatively small number of ESTs has provided sufficient information to engage in further molecular, physiological and genetic studies. For instance, the pretreatment with salicylate likely enriched the expression of pathogenesis-related transcripts that can now be used to study disease progression in specific strawberry cultivars with large variations in sensitivity and resistance. Clearly, the development of a comprehensive SSR catalog allows characterization of these potential genetic markers in the progeny of polymorphic cultivars, in an important crop species virtually devoid of linkage associations. Unlike other markers, EST-derived SSRs by definition originate from a sequence that is expressed, adding functional resolution to linkage groups built on structural polymorphisms. More importantly, the same suite of tools used to perform these analyses will be soon available through a public interface at the GDR, making comparable analyses possible. These applications are an important rationale for sequencing and analysis of a limited EST set, as even a small research program may find sufficient resources to initiate molecular-genetic study of an under-represented crop species.

## Methods

### Library construction

Roots, leaves, petioles, stolons, meristems and new daughter plants were harvested from several individual chamber-grown strawberry (*Fragari*a × *ananassa *cultivar 'Strawberry Festival) plants 24 h after salicylic acid treatment (4 μm foliar spray, 1 μM drench). Tissues were washed briefly to remove soil and then were frozen in liquid nitrogen. Total RNA was extracted using the following method, a modification of protocols used in the extraction of RNA from pine cones [34]. Briefly, 1 g of tissue was ground in liquid nitrogen using a mortar and pestle, then incubated in extraction buffer (2% CTAB, 2% polyvinylpyrrolidone, 100 mM Tris-HCl (pH 8.0), 25 mM EDTA, 2.0 M NaCl, 0.5 g/ml spermidine, and 2.0% β-mercaptoethanol) at 65°C for 10 min. The samples were cooled to room temperature, an equal volume of chloroform:octonol (24:1) was added and the mixture was homogenized using a Polytron (T10-35 homogenizer) at 80–90% maximum speed. The organic and aqueous phases were separated by centrifugation at 5700 × g and the supernatant was vortexed with an equal volume of chloroform:octonol. The phases were again separated by centrifugation and the supernatant was transferred to a clean test tube, LiCl was added to a final concentration of 2.5 M and precipitated on ice overnight. RNA was then collected by centrifugation at 5700 × g. The pellet was resuspended in 500 μl SSTE (1 M NaCl, 0.5% SDS, 10 mM Tris-HCl (pH 8.0), 1 mM EDTA) and extracted with an equal volume of chloroform:octonol. The supernatant was precipitated with two volumes of ethanol, the pellet was washed with 76% ethanol containing 0.3 M sodium acetate, dried briefly in a Speed Vac, and resuspended in 50 μl 10 mM Tris-HCl (pH 8.0) 2.5 mM EDTA before quantitation by spectrophotometry.

For library construction mRNA was isolated from total RNA using the Oligotex Direct mRNA Mini Kit (Qiagen Inc., Valencia, CA) using 500 μg total RNA. The cDNA library was constructed from 5 μg mRNA using the Uni-ZAP XR Cloning Kit (Stratagene Inc, Carlsbad, CA) as per manufacturer's directions. The primary library consisted of 6.2 × 10^7 ^colony forming units with average insert size of 800 bp and 98% of clones containing inserts of ≥ 200 bp. Mass excision of filamentous phage was performed and phagemids were cloned to E. coli for sequencing.

### Sequencing and sequence processing

A total of 1847 EST clones were sequenced from the 3' end at the University of Florida ICBR Core Facility using ET Terminator (Amersham Inc, Schaumburg, IL). These sequences were processed using publicly available software incorporated in a fully automated in-house script (ProcEST.pl) developed at Clemson University by the Genome Database for Rosaceae (GDR) bioinformatics team. Sequence trace files were converted into FASTA formatted sequence and quality score files using the PHRED [35] base-calling program. Vector and host contamination were identified and masked using the sequence comparison program CROSS_MATCH [36]. Vector trimming excised the longest non vector sequence and further trimming removed low quality bases (less than phred score 20) at both ends of a read. Sequences were discarded if they had greater than 5% ambiguous bases, more than 40 PolyA or Poly T bases or less than 100 high quality bases (minimum phred score of 20). Using this protocol, 81% of the sequences (1505) were considered high quality and submitted to the NCBI public EST repository dbEST [37]. To reduce redundancy and increase transcript length the high quality sequences were assembled using the contig assembly program CAP3 [38]. Various assemblies were performed using different CAP3 parameters to identify the build that required least manual editing. More stringent parameters (- p 90 -d 60) were used to prevent over assembly and help identify potential paralogs. The assembly was refined where possible using homology to the SwissProt database to indicate contig accuracy. Homology was determined by comparing the contigs and clones against the Swiss Prot database using the fastx3.4 algorithm [7] with EXP < 1e -6. Contigs whose clones showed difference in homology were deconstructed and contigs with the same homology to other contigs were joined using default CAP3 parameters. The unigene data set was derived by combining the contig and singleton data sets.

### Functional characterization

Functional characterization of the unigene data set consisted of pairwise comparison of both the high quality clones and the contig consensus sequences against the NCBI nr [39] and SWISS-PROT [40] protein databases using the fastx3.4 algorithm [7]. The most significant matches (EXP < 1e ^-7 ^and EXP <1e^-6 ^for the NCBI nr SWISS-PROT searches, respectively) for each contig and individual clones in the library were recorded. The Swiss-Prot matches were further classified by gene ontology [41]. Contigs or clones that did not have a significant match with either of these databases were searched against the InterPro protein families and domains database (Mulder et al, 2005) using InterProScan [42].

The unigene sequences were also characterized by comparison with the Genbank Rosaceae EST dataset (227250 as of February 14, 2005) and 256 peach mapped ESTs [43], downloaded from the Genome Database for Rosaceae (GDR). Using the BLASTN algorithm [44], sequences with > 85% similarity over an alignment length of 100 bp were considered significant matches.

### Open reading frame and microsatellite analysis

Open reading frames (ORFs) were identified in the ESTs using the software program FLIP [45] and the longest ORF was recorded as the putative coding region. Simple Sequence Repeats (SSRs) were identified in the unigene data set using a modified version (CUGISSR) of a perl script SSRIT [46]. SSRs recorded for the final dataset include dimers with at least 5 repeats, trimers with at least 4 repeats, tetramers with at least 3 repeats, and pentamers with at least 3 repeats. SSR-containing sequences were identified as optimal candidates for primer development if they contained a GC content between 40% and 60% and a minimum of 20 base pairs of sequence on either side of the SSR. Using the FLIP output, CUGISSR reports the location of SSRs in the relation to the putative coding region.

### Data storage and web interface

All sequence, assembly, homology, ORF and SSR data were uploaded to the Genome Database for Rosaceae (GDR) (Jung et al, 2004) as well as library, protocol, contact and publication information. GDR scripts were utilized to allow users to browse, query or download all the project data.

### Public access and dissemination

The GDR website has a number of different EST project sections including the Fragaria EST dataset detailed here. These web pages are extensively linked such that users can easily access data of interest regardless of the navigation entry point. To access the project pages for this EST project, users can go to the project page which can be found by a link in the "About Us" drop down menu in the top navigation bar. This project is listed on the "Data Overview" page as "Folta – University of Florida" [47]. The sidebar for this project allows the user to view the project description, the library details, the processing protocol, a report on the successful clones, unigene details, gene homology pages, microsatellite analysis, contact information, and publication information. The cDNA phage library and individual clones generated in this study are available upon request.

For members of the Rosaceae community or of the public who are interested in searching the dataset, the EST search page allows users to search the *Fragaria *sequence set directly [48]. The ESTs and the unigene can be searched by name, by homology, and by features such as presence of a microsatellite or component of a contig. Once an EST or contig has been selected, the sidebar allows users to view all information relating to the sequence (or consensus sequence), the library details, the assembly information, the open reading frame and microsatellites, homology, and for contigs, the component ESTs.

## Authors' contributions

KF prepared the RNA for, and generated the cDNA libraries, provided functional annotation and analysis and drafted the manuscript with MS. MS and CJ performed all computational analyses under the guidance of SJ and DM. PS and DB collected plant tissue, participated in RNA isolation and functional EST annotation. All authors read and approved the final manuscript.

## Figures and Tables

**Table 1:** Homology between the *Fragaria *× *ananassa *unigene sequences in public database as inferred through comparisons to Genbank nr, SWISSPROT, Rosaceae ESTs and mapped peach ESTs.

	**nr.pep.1^1^**	**sprot.fas^1^**	**Rosaceae ESTs^2^**	**mapped peach ESTs^2^**
**database size**	2321663	153871	227250	256
**total # sequences**	1304	1304	1304	1304
**# sequences with matches**	1105	714	835	22
**% sequences with matches**	84.74%	54.75%	64.03%	1.69%
**# sequences w/o matches**	199	590	469	1282
**% sequences w/o matches**	15.26%	45.25%	35.97%	98.31%

^1 ^FASTX3.4 Algorithm with cut-off of < 1e^-6^

^2 ^BLASTN Algorithm with identity >= 85% and overlap > = 100

**Table 2:** The summarized results of homology searches of the *Fragaria *× *ananassa *unigene sequences against SPROT, nr pep and Rosaceae ESTs.

	**Number**	**Percent**
**Sequences with hits to sprot**	714	54.75%
**Sequences without hits to sprot**	590	45.25%
**Sequences without hits to sprot with hits to nr.pep**	399	30.60%
**Sequences without hits to sprot or nr.pep**	191	14.64%
**Sequences without hits to sprot or nr.pep but with hits to Rosaceae**	54	4.14%
**Sequences without hits to any database**	137	10.51%

**Table 3:** *Fragaria *ESTs with homology to mapped peach BACs

**Strawberry Unigene**	**Peach EST**	**GenBank Accession of Peach EST**	**Homology**	**E-Value from BLAST output**
Contig 25	PP_LEa0003M24f	BU039764	enolase 2 (allergen HEVb8)	1e^-111^
Contig 37	PP_LEa0003O13f	BU039800	ubiquitin extension protein	6e^-33^
Contig 64	PP_LEa0003O13f	BU039800	ubiquitin extension protein	3e^-51^
Contig 131	PP_LEa0003M11f	BU039753	glyceraldehyde-3-phosphate dehydrogenase	0.0
FA_SEa0001D01r	PP_LEa0009G24f	BU041453	ion stress related protein	2e^-39^
FA_SEa0003G08r	PP_LEa0027M15f	BU046817	20S proteosome subunit PAF1	1e-^166^
FA_SEa0006D03r	PP_LEa0011D12f	BU042012	ribosomal protein L7	2e^-34^
FA_SEa0007B10r	PP_LEa0003O13f	BU039800	ubiquitin extension protein	8e^-35^
FA_SEa0008B09r	PP_LEa0012A24f	BU042298	expressed protein	1e^-78^
FA_SEa0008C05r	PP_LEa0004I18f	BU039998	cysteine protease	1e^-81^
FA_SEa0009F12r	PP_LEa0035B03f	BU048314	allergen PRU AV 1	4e^-32^
FA_SEa0011D12r	PP_LEa0003O13f	BU039800	ubiquitin extension protein	4e^-66^
FA_SEa0011E01r	PP_LEa0009N05f	BU041589	expressed protein	4e^-63^
FA_SEa0011F03r	PP_LEa0011F03f	BU042049	Centrin	1e^-74^
FA_SEa0012D04r	PP_LEa0036C16f	BU048550	putative gsh-dependent dehydroascorabate reductase 1	1e^-144^
FA_SEa0015A08r	PP_LEa0006B19f	BU040484	heat shock protein 70	0.0
FA_SEa0015F10r	PP_LEa0003P11f	BU039816	cytosolic aldolase	2e^-30^
FA_SEa0015H12r	PP_LEa0013C06f	BU042571	cell switch protein	e^-177^
FA_SEa0016A10r	PP_LEa0035H24f	BU048407	putative ribosomal protein	9e^-64^
FA_SEa0018A02r	PP_LEa0027P18f	BU046858	ADP-ribosylation factor	0.0
FA_SEa0018D07r	PP_LEa0036E14f	BU048583	putative glyoxylase II	3e^-47^
FA_SEa0018F08r	PP_LEa0035M02f	BU048454	NADH-cytochrome b5 reductase	2e^-61^

**Table 4:** The frequency of simple sequence repeats in the *Fragaria *unigene.

**Motif Length**	**Frequency**	**Percentage Frequency**
2 bp	88	32.7%
3 bp	102	36.8%
4 bp	75	27.9%
5 bp	7	2.6%

**Table 5:** Frequency of different types of dinucleotide and trinucleotide repeats in the *Fragaria *unigene set

**Motif**	**Frequency**	**Percentage Frequency**
AT/TA	15	5.58%
AG/GA/CT/TC	65	24.16%
AC/CA/TG/GT	8	2.97%
GC/CG	0	0.00%
AAT/ATA/TAA/ATT/TTA/TAT	2	0.74%
AAG/AGA/GAA/CTT/TTC/TCT	35	13.01%
AAC/ACA/CAA/GTT/TTG/TGT	4	1.49%
ATG/TGA/GAT/CAT/ATC/TCA	6	2.23%
AGT/GTA/TAG/ACT/CTA/TAC	0	0.00%
AGG/GGA/GAG/CCT/CTC/TCC	21	7.81%
AGC/GCA/CAG/GCT/CTG/TGC	10	3.72%
ACG/CGA/GAC/CGT/GTC/TCG	7	2.60%
ACC/CCA/CAC/GGT/GTG/TGG	10	3.72%
GGC/GCG/CGG/GCC/CCG/CGC	7	2.60%

**Table 6:** Statistics for optimal primer candidates

	**Inside ORF**	**Outside ORF**
2 bp	35 (24.6%)	21 (31.8%)
3 bp	77 (54.2%)	14 (21.2%)
4 bp	29 (20.4%)	26 (39.4%)
5 bp	1 (0.7%)	5 (7.6%)
Total	142 (68.3%)	66 (31.7%)

**Table 7:** Transcripts corresponding to genes of described function in important physiological processes

**EST**	**Homolog**	**E**
**Photoperiodic Control of Flowering Time**		

FA_Sea0007C05	B-box, zinc-finger protein CONSTANS	7.30E-21
FA_Sea0020G05	B-box, zinc-finger protein CONSTANS	3.40E-06
FA_Sea0016A05	MADS box protein AGL20/SUPPRESSOR OF CONSTANS	1.90E-15
FA_Sea0002H08	VIN3 – Vernalization insensitive 3 protein	1.50E-33

**Disease Resistance**		

FA_Sea0004D05	Disease resistance protein (TIR-NBS-LRR class)	6.80E-18
FA_Sea0006F10	Enhanced Disease Susceptibility protein EDS5	7.10E-58
FA_Sea0007F04	Plant defensin PDF2.2	3.10E-22
FA_Sea0010B10	Pathogenesis-related thaumatin (PR5)	1.20E-21
FA_Sea0014H12	Putative thaumatin (PR5)	7.10E-16
FA_Sea0015A01	Harpin-induced protein	3.80E-26
FA_Sea0015D01	NDR1 family protein	7.00E-69
FA_Sea0017F09	Disease resistance protein (CC-NBS-LRR class)	2.10E-23
FA_SEa0020H01	Harpin-induced protein	3.40E-18
FA_Sea0010F01	glycosyl hydrolase family 17 p (PR2)	2.60E-12
FA_Sea0017H06	Osmotin-like protein (PR5)	5.00E-13
FA_SEa0001D03	Peroxidase PRXR1 (PR9)	8.20E-51
FA_SEa0019D07	Bet v 1 (PR10)	2.10E-35
FA_SEa0012C06	Lipid transfer protein LPT4 (PR14)	1.70E-19

**Photomorphogenesis**		

FA_Sea0004E09	B-zip transcription factor HY5	4.60E-37
FA_Sea0001C09	NON-PHOTOTROPIC HYPOCOTYL 3	3.20E-31
FA_SEa0006H04	Far-red impaired / FAR1	3.30E-29
